# Urinary cell-free nucleic acid IQGAP3: a new non-invasive diagnostic marker for bladder cancer

**DOI:** 10.18632/oncotarget.24436

**Published:** 2018-02-07

**Authors:** Won Tae Kim, Ye Hwan Kim, Pildu Jeong, Sung-Pil Seo, Ho-Won Kang, Yong-June Kim, Seok Joong Yun, Sang-Cheol Lee, Sung-Kwon Moon, Yung-Hyun Choi, Geun Taek Lee, Isaac Yi Kim, Wun-Jae Kim

**Affiliations:** ^1^ Department of Urology, Chungbuk National University College of Medicine, Cheongju, Chungbuk, South Korea; ^2^ Department of Urology, Chungbuk National University Hospital, Cheongju, Chungbuk, South Korea; ^3^ School of Food Science and Technology, Chung-Ang University, Anseong, South Korea; ^4^ Department of Biochemistry, Dongeui University College of Oriental Medicine, Busan, South Korea; ^5^ Section of Urological Oncology, The Cancer Institute of New Jersey, Robert Wood Johnson Medical School, New Brunswick, NJ, USA

**Keywords:** biomarkers, nucleic acids, urinary bladder neoplasms, urine

## Abstract

**Background:**

There is growing interest in developing new non-invasive diagnostic tools for bladder cancer (BC) that have better sensitivity and specificity than cystoscopy and cytology. This study examined the value of urinary cell-free nucleic acid (NA) as a diagnostic marker for BC.

**Material and methods:**

A total of 81 patients (74 BC and 7 normal controls) were used for a tissue set, and 212 patients (92 BC and 120 normal controls) were used as a urine set. Expression of tissue mRNA and urinary cell-free NAs was then examined.

**Results:**

Four candidate genes were top-ranked in the tissue microarray. Expression levels of two of these (IQGAP3 and TOP2A) in BC tissue and urine samples from BC patients were significantly higher than those in samples from the control groups. Binary logistic regression analysis of cell-free NA levels in urine samples revealed that IQGAP3 was significantly associated with BC: PicoGreen-adjusted odds ratio (OR), 3.434; confidence interval (CI), 2.999–4.180; *P*<0.001; RiboGreen-adjusted OR, 2.242; CI, 1.793–2.840; *P*<0.001. Further analysis of IQGAP3 urinary cell-free NAs with respect to tumor invasiveness and grade also yielded a high AUC, suggesting that IQGAP3 can discriminate between BC patients and non-cancer patients with hematuria.

**Conclusions:**

Levels of IQGAP3 urinary cell-free NA in BC patients were significantly higher than those in normal controls or patients with hematuria. High levels of IQGAP3 urinary cell-free NA also reflected high expression in BC tissues. Therefore, IQGAP3 urinary cell-free NA may be a complementary diagnostic biomarker for BC.

## INTRODUCTION

Bladder cancer (BC) is the ninth most common cancer worldwide [1]. In 2012, an estimated 430,000 new cases were diagnosed globally. The highest incidence rates are observed in North America, Southern/Western Europe, Northern Africa, and Western Asia. Although the mortality rates in the most developed countries have fallen, BC ranks 13^th^ in terms of global cancer-related deaths [1].

More than 90% of BCs comprise transitional cell carcinoma, and most of these are papillary non-muscle invasive BC (NMIBC) [2]. However, approximately 20% of NMIBCs progress to muscle invasive BC (MIBC); 25% of newly diagnosed BC patients have MIBC. At the time of diagnosis, nearly 50% of MIBC cases have occult distant metastases [2]; thus early diagnosis of BC is very important.

At the present time, the gold standard diagnostic methods for BC are cystoscopy and urine cytology. However, urine cytology has poor sensitivity (except for high grade tumors) [3] and, although flexible cystoscopy has been introduced recently, the procedure is both invasive and uncomfortable [4]. To solve these problems, a number of urine-based diagnostic markers, such as bladder tumor antigen, nuclear matrix protein 22 (NMP22), and fluorescence *in situ* hybridization (FISH), have been developed [5, 6]. Unfortunately, none are superior to cystoscopy and cytology. Therefore, there is growing interest in new non-invasive diagnostic tools that have better sensitivity and specificity for BC.

**Figure 1:**
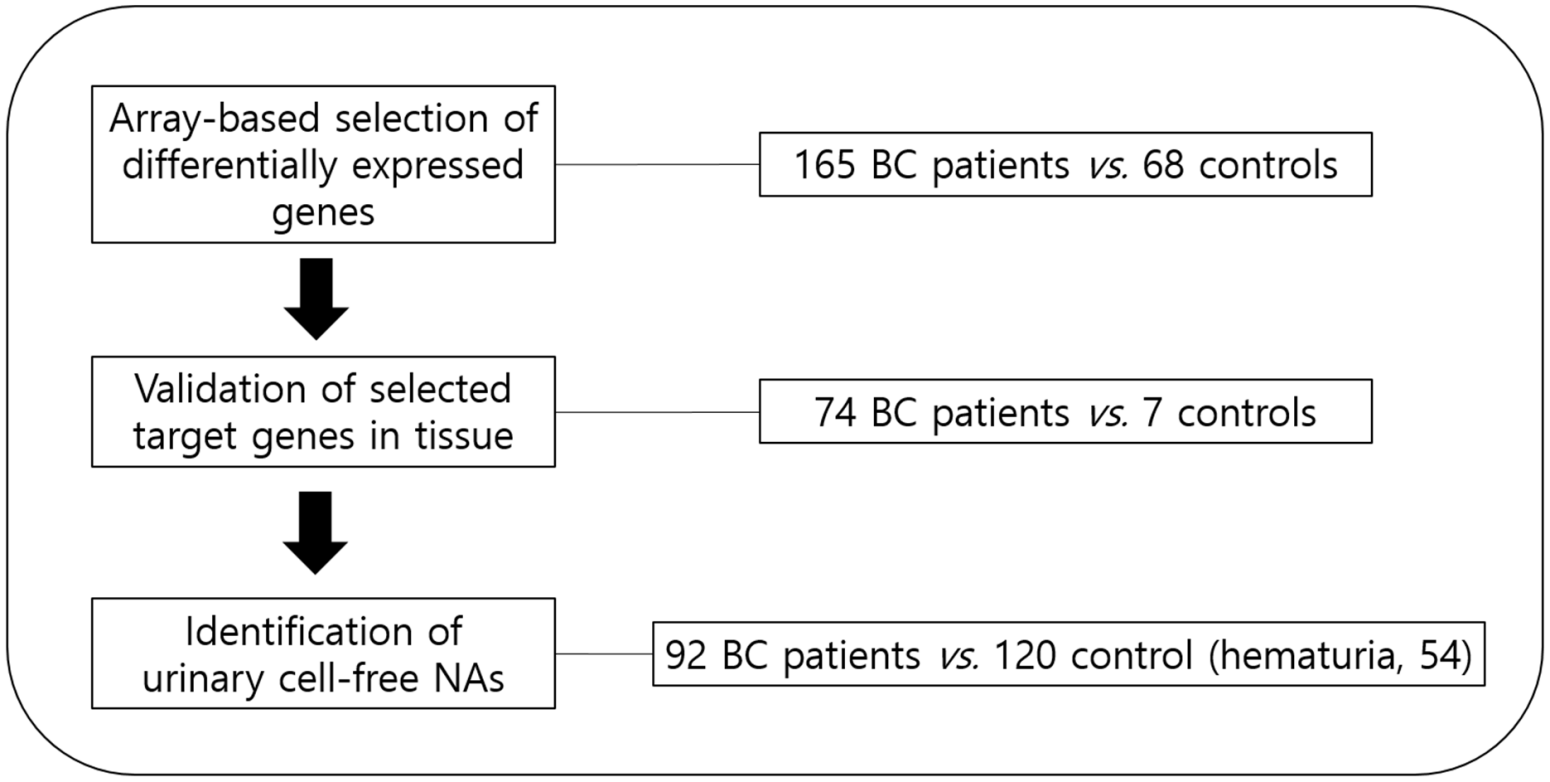
Schematic showing the study design

Several reports and reviews have examined/discussed urinary nucleic acids (NAs) [7–9]. The main source of urinary cell-free NAs is thought to be apoptotic and necrotic cancer cells [9]. Urinary cell-free NAs might be gathered as a result of renal cell-free NA transport from the blood or direct contact from urinary tracts. Most of the cancer-specific cell-free NA present in the urine of patients with urinary tract cancers is not derived from the blood; rather, it is derived directly from the tumor cells [9]. Therefore, the main aims of the present study were to identify candidate tissue mRNAs from BC microarrays, examine differences in expression of these candidate mRNAs in tissue from BC patients and controls, and investigate the levels of selected cell-free NAs in urine samples from BC patients and normal or hematuria controls (Figure 1). The overall aim was to assess the value of urinary cell-free NA as a diagnostic marker for BC.

## RESULTS

### Baseline characteristics of the study patients in the tissue and urine sets

The baseline characteristics of all patients and controls are shown in Table 1.

**Table 1:** Baseline characteristics of the patients in the tissue and urine sets

Variable (%)	Tissue set		Urine set			
	BC cases	Controls	BC cases	Controls	Healthy controls	Hematuria controls
Number	74	7	92	120	66	54
Mean age (y) ± SD	65.26 ± 13.87	50.14 ± 10.43	65.62 ± 12.89	66.72 ± 9.63	68.77 ± 8.06	64.22 ± 10.82
Gender						
Male	59 (79.7)	1 (14.3)	73 (79.3)	98 (81.7)	61 (92.4)	37 (68.5)
Female	15 (20.3)	6 (85.7)	19 (20.7)	22 (18.3)	5 (7.6)	17 (31.5)
Grade						
G1	17 (23.0)		19 (20.7)			
G2	34 (45.9)		41 (44.6)			
G3	23 (31.1)		32 (34.8)			
T stage						
Ta	16 (21.6)		22 (23.9)			
T1	32 (43.3)		36 (39.1)			
T2	6 (8.1)		8 (8.7)			
T3	8 (10.8)		7 (7.6)			
T4	12 (16.2)		19 (20.7)			
N stage						
N0	65 (87.8)		80 (87.0)			
N (1–3)	9 (12.2)		12 (13.0)			
M stage						
M0	67 (90.5)		85 (92.4)			
M1	7 (9.5)		7 (7.6)			

BC, bladder cancer.

### Validation of BC tissue candidate mRNA identified from microarray data

**Figure 2:**
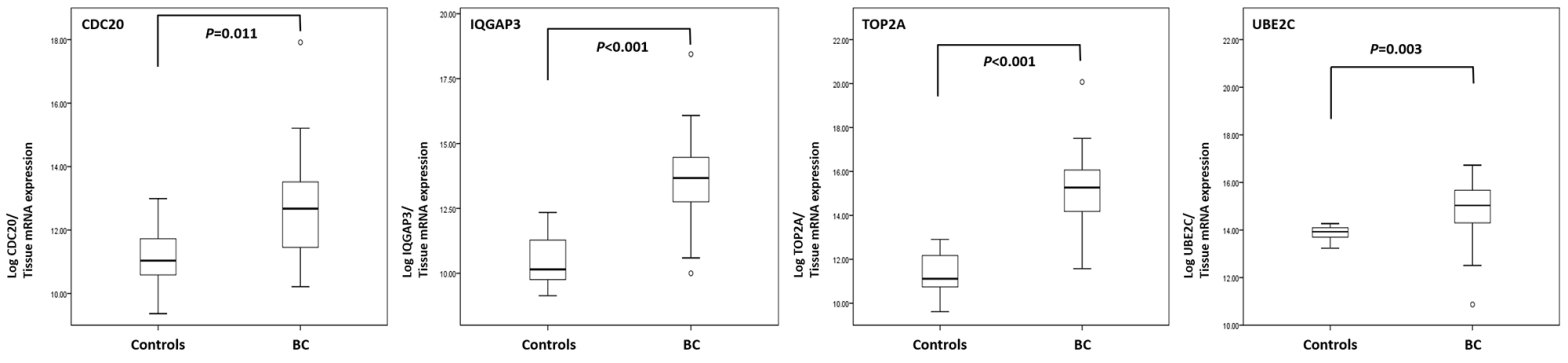
Comparison of gene expression levels in tissue samples from bladder cancer patients and normal controls Real-time PCR analysis of CDC20, IQGAP3, TOP2A, and UBE2C mRNA expression in BC and control tissues (*P*=0.011, *P*<0.001, *P*<0.001, and *P*=0.003, respectively; Mann-Whitney U test). BC, bladder cancer.

Table 2 lists the four candidate tissue mRNAs selected from tissue mRNA array data derived from BC patients and normal controls. Four candidate genes were identified as top-ranked based on their increased expression in BC tissues compared with that in normal controls. Expression of cell-division cycle protein 20 (CDC20), isoleucine glutamine motif-containing GTAase-activating proteins (IQGAP3), DNA topoisomerase 2-alpha (TOP2A), and ubiquitin-conjugating enzyme E2 C (UBE2C) in BC tissue samples from BC patients was significantly higher than that in tissue samples from controls (*P*=0.011, *P*<0.001, *P*<0.001, and *P*=0.003, respectively) (Table 2 and Figure 2). IQGAP3 and TOP2A were selected as candidate markers for BC detection because the difference in expression between patients and controls was the most significant.

**Table 2:** Candidate mRNAs in tissues^*^

Gene symbol	Tissue mRNA array	Tissue real-time PCR expression data			
	(normal *vs*. BC)		Normal controls	BC	
	*P*-value	-fold change	(expression level, × 10^4^ copies/μg) Median (IQR)^a^	(expression level, × 10^4^ copies/μg) Median (IQR)^a^	*P*-value^b^
CDC20	0.001	3.9323197	6.2 (2.8–19.8)	32.0 (9.0–77.2)	0.011
IQGAP3	0.001	3.3673384	2.6 (1.6–10.4)	86.6 (32.6–201.9)	<0.001
TOP2A	0.001	3.2166836	6.7 (3.8–24.6)	428.0 (143.3–1030.3)	<0.001
UBE2C	0.001	2.9033677	111.9 (82.8–140.7)	340.1 (161.5–643.6)	0.003

^*^ These tissue mRNAs were chosen because they showed the greatest increase in expression in bladder cancer tissue compared with control tissue in microarrays.

IQR, interquartile range; BC, bladder cancer.

^a^ Copy number of corresponding transcripts (per μg of total RNA) used for cDNA synthesis.

^b^ Mann-Whitney U test.

### Expression of urinary cell-free NAs in BC patients and normal controls

**Figure 3:**
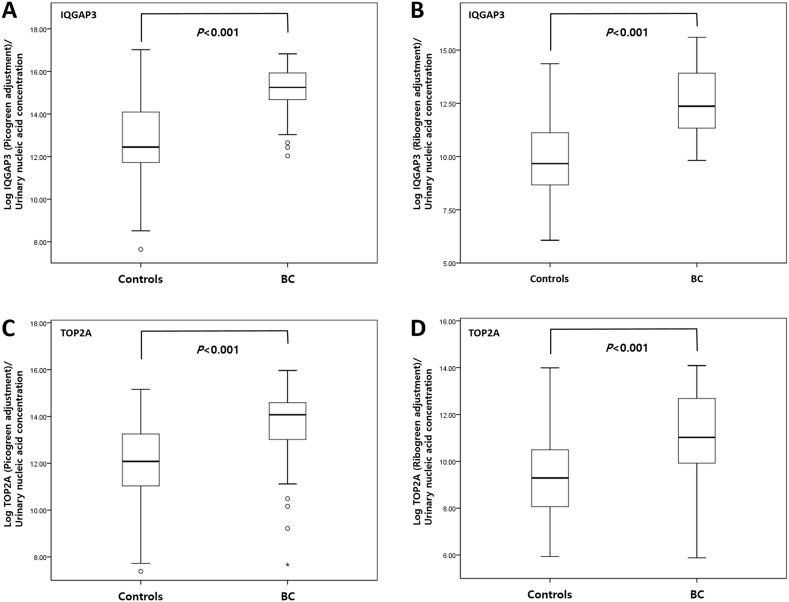
Comparison of urinary cell-free NA levels in samples from bladder cancer patients and normal controls Real-time PCR analysis of urinary cell-free NAs IQGAP3 **(A, B)** and TOP2A **(C, D)** was performed using PicoGreen (A, C) and RiboGreen (B, D) (all *P*<0.001; Mann-Whitney U test). BC, bladder cancer.

As shown in Figure 3, the levels of NAs of IQGAP3 and TOP2A in urine samples from BC patients were significantly higher than those in samples from normal controls (each *P*<0.001) (Table 3). In particular, the selected NAs were significantly higher in cancer patients than controls after PicoGreen and RiboGreen adjustment.

**Table 3:** Levels of urinary cell-free nucleic acid in BC and normal controls

Urinary cell-free nucleic acids	Normal controls		BC	*P*-value^b^
	**(expression levels, × 10^3^ copies/μg) Median (IQR)^a^**	**(expression levels, × 10^3^ copies/μg) Median (IQR)^a^**
PicoGreen adjustment	IQGAP3	223.6 (119.6–1317.5)	4200.5 (2322.7–8348.6)	<0.001
	TOP2A	175.8 (60.8–572.7)	1293.8 (446.4–2178.1)	<0.001
RiboGreen adjustment	IQGAP3	9.8 (3.3–56.3)	221.9 (69.2–1109.0)	<0.001
	TOP2A	9.5 (2.2–33.6)	61.3 (20.3–325.0)	<0.001

IQR, interquartile range.

^a^ Copy number of corresponding transcripts (per μg of total nucleic acids).

^b^ Mann-Whitney U test; BC, bladder cancer.

### Selection of candidate urinary cell-free NAs by ROC analysis

**Figure 4:**
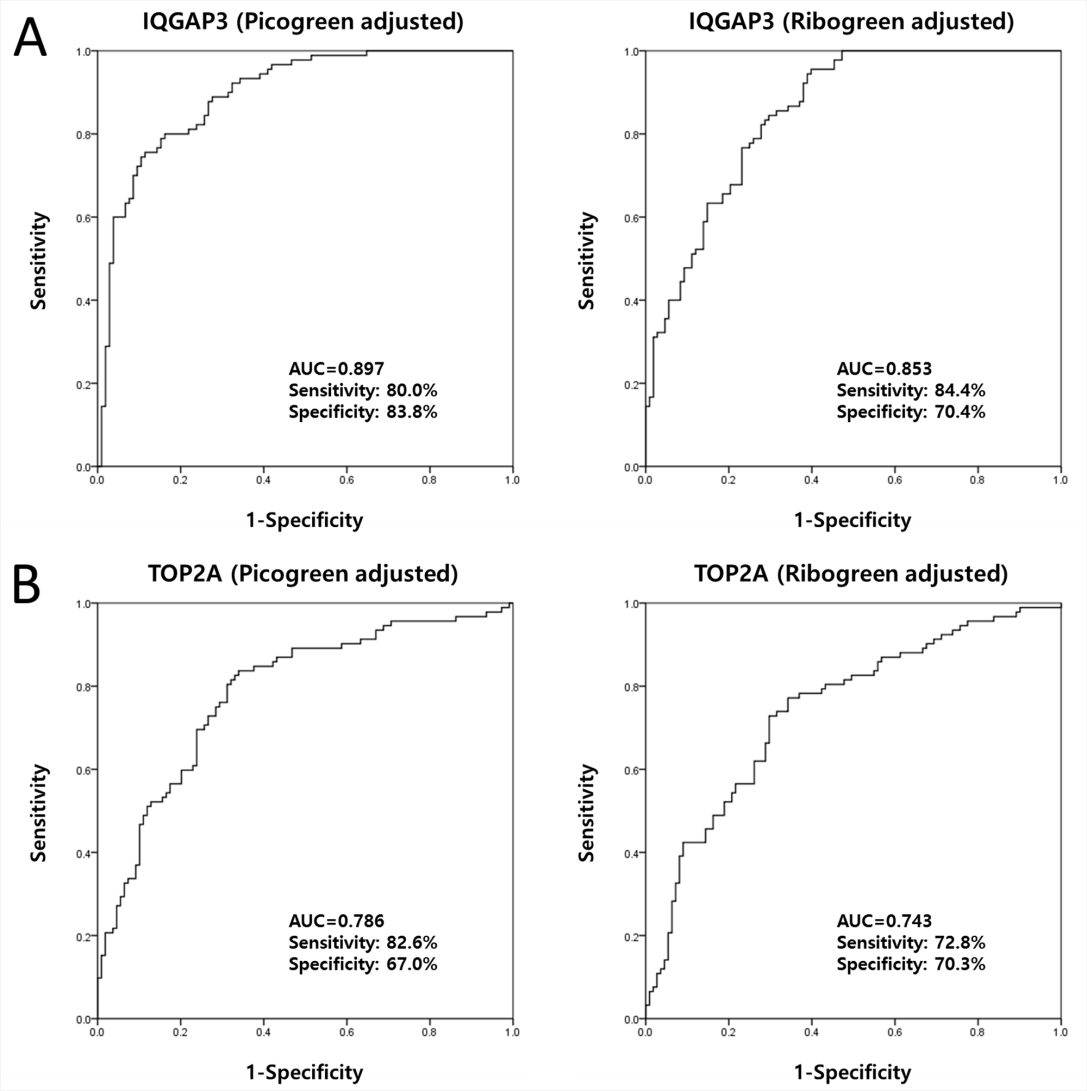
Receiver operating characteristic curve analysis of candidate urinary cell-free NAs selected from tissue mRNA expression data The AUCs for IQGAP3 were 0.897 (PicoGreen-adjusted) and 0.853 (RiboGreen-adjusted) **(A)**. The AUCs for TOP2A were 0.786 (PicoGreen-adjusted) and 0.743 (RiboGreen-adjusted) **(B)**. AUC, area under the curve.

ROC curve analysis revealed that the area under the curve (AUC) for IQGAP3 urinary cell-free NA was above the selected cut-off value of 0.8 (the AUCs for IQGAP3 after PicoGreen and RiboGreen adjustment were 0.897 and 0.853, respectively) (Figure 4). The cut-off values for PicoGreen- and RiboGreen-adjusted IQGAP3 (14.5156 and 10.7485, respectively) that yielded the highest combined sensitivity (80.0% and 84.4%, respectively) and specificity (83.8% and 70.4%, respectively) for BC detection were selected for further analysis.

### IQGAP3 as a diagnostic marker that differentiates between BC and hematuria

**Figure 5:**
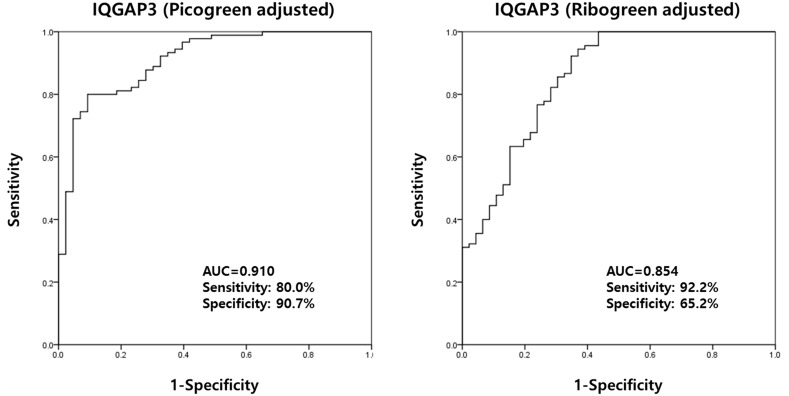
Receiver operating characteristics curve analysis of IQGAP3 urinary cell-free NA levels in bladder cancer patients and hematuria controls The AUC for IQGAP3 was 0.910 (PicoGreen-adjusted) and 0.854 (RiboGreen-adjusted) (comparison made between BC patients and hematuria controls). AUC, area under the curve.

Urinary levels of IQGAP3 showed good utility as a diagnostic marker that can differentiate between BC patients and non-cancer patients with hematuria (PicoGreen-adjusted AUC, 0.910; sensitivity, 80.0%; specificity, 90.7%; RiboGreen-adjusted AUC, 0.854; sensitivity, 92.2%; specificity, 65.2%) (Figure 5).

### Binary logistic regression analysis to confirm urinary cell-free NAs as markers of BC

Binary logistic regression analysis of cell-free NA levels in urine samples revealed that PicoGreen-adjusted and RiboGreen-adjusted IQGAP3 levels were significantly associated with BC (odds ratio (OR), 3.434; confidence interval (CI), 2.999–4.180; *P*<0.001 and OR, 2.242; CI, 1.793–2.840; *P*<0.001, respectively) (Table 4). These data suggest that urinary IQGAP3 levels can discriminate BC patients from patients with hematuria and healthy controls.

**Table 4:** Binary logistic regression analysis of urinary IQGAP3 cell-free nucleic acids as a biomarker for bladder cancer

Urinary cell-free nucleic acids	BC	
(IQGAP3)	OR (95% CI)	*P*-value
PicoGreen adjustment		
*vs*. normal controls	3.434 (2.459–4.795)	<0.001
*vs*. hematuria controls	3.740 (2.430–5.755)	<0.001
RiboGreen adjustment		
*vs*. normal controls	2.242 (1.793–2.840)	<0.001
*vs*. hematuria controls	2.384 (1.770–3.212)	<0.001

OR, odds ratio; CI, confidence interval.

BC, bladder cancer.

### IQGAP3 is a valuable diagnostic marker for both NMIBC and MIBC

**Figure 6:**
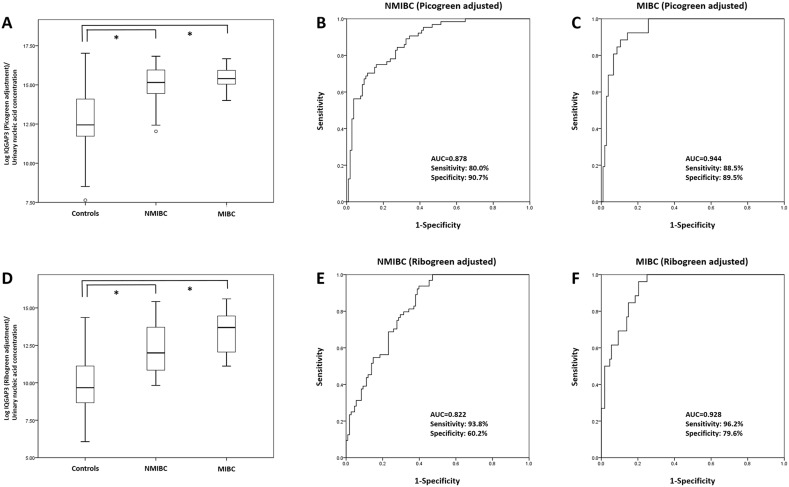
Receiver operating characteristics curve analysis of IQGAP3 urinary cell-free NA expression in NMIBC and MIBC Expression of IQGAP3 urinary cell-free NA in NMIBC and MIBC patients was significantly higher than that in normal controls (PicoGreen- and RiboGreen-adjusted groups; each *P*<0.001) **(A, D)**. In the PicoGreen-adjusted group, the AUC values for NMIBC and MIBC were 0.878 and 0.949, respectively **(B, C)**. In the RiboGreen-adjusted group, the AUC values for NMIBC and MIBC were 0.822 and 0.9928, respectively **(E, F)**. AUC, area under the curve; NMIBC, non-muscle invasive bladder cancer; MIBC, muscle invasive bladder cancer.

As shown in Figure 2, levels of IQGAP3 urinary cell-free NA in samples from both NMIBC and MIBC patients were significantly higher than those from normal controls (*P*<0.001, each) (Table 5). Binary logistic regression analysis revealed that IQGAP3 urinary NA levels were significantly associated with NMIBC (PicoGreen adjustment: OR, 2.999; CI, 2.152–4.180; *P*<0.001; RiboGreen adjustment: OR, 2.016; CI, 1.609–2.524; *P*<0.001) and MIBC (PicoGreen adjustment: OR, 6.416; CI: 2.866–14.365; *P*<0.001; RiboGreen adjustment: OR, 2.977; CI, 1.982–4.470; *P*<0.001) (Table 6). ROC curve analysis revealed that the PicoGreen-adjusted AUCs for IQGAP3 urinary cell-free NAs in NMIBC and MIBC and the RiboGreen-adjusted AUCs for IQGAP3 in NMIBC and MIBC were 0.878 and 0.944, respectively, and 0.822 and 0.928, respectively (Figure 6). The highest combined sensitivity of IQGAP3 for NMIBC and MIBC was 80% (PicoGreen-adjusted) and 93.8% (RiboGreen-adjusted) and 88.5% (PicoGreen-adjusted) and 96.2% (RiboGreen-adjusted), respectively, whereas the greatest specificity was 90.7% (PicoGreen-adjusted) and 60.2% (RiboGreen-adjusted) and 89.5% (PicoGreen-adjusted) and 79.6% (RiboGreen-adjusted), respectively.

**Table 5:** Levels of IQGAP3 urinary cell-free nucleic acids in NMIBC, MIBC, and normal controls

Urinary cell-free nucleic acids	Normal controls	NMIBC		MIBC	
**(expression levels, × 10^3^ copies/μg) Median (IQR)^a^**	**(expression levels, × 10^3^ copies/μg) Median (IQR)^a^**	***P*-value^b^**	**(expression levels, × 10^3^ copies/μg) Median (IQR)^a^**	***P*-value^b^**
PicoGreen adjustment	223.6 (119.6–1317.5)	3813.0 (1816.7–8499.3)	<0.001	4873.3 (3407.7–8632.2)	<0.001
RiboGreen adjustment	9.8 (3.3–56.3)	153.2 (46.5–874.1)	<0.001	893.5 (167.5–1956.7)	<0.001

IQR, interquartile range; NMIBC, non-muscle invasive bladder cancer; MIBC, muscle invasive bladder cancer.

^a^ Copy number of corresponding transcripts (per μg of total nucleic acids).

^b^ Mann-Whitney U test.

**Table 6:** Binary logistic regression analysis of urinary IQGAP3 cell-free nucleic acids as a biomarker for NMIBC and MIBC

Urinary cell-free nucleic acids	NMIBC		MIBC	
**(IQGAP3)**	**OR (95% CI)**	***P*-value**	**OR (95% CI)**	***P*-value**
PicoGreen adjustment				
*vs*. normal controls	2.999 (2.152–4.180)	<0.001	6.416 (2.866–14.365)	<0.001
*vs*. hematuria controls	3.280 (2.141–5.025)	<0.001	12.654 (3.098–51.686)	<0.001
RiboGreen adjustment				
*vs*. normal controls	2.016 (1.609–2.524)	<0.001	2.977 (1.982–4.470)	<0.001
*vs*. hematuria controls	2.153 (1.601–2.895)	<0.001	3.084 (1.828–5.204)	<0.001

OR, odds ratio; CI, confidence interval.

NMIBC, non-muscle invasive bladder cancer; MIBC, muscle invasive bladder cancer.

### Urinary levels of IQGAP3 according to tumor grade

**Figure 7:**
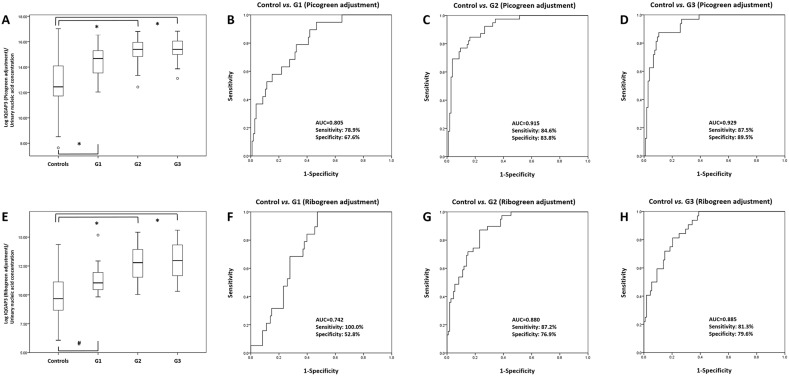
Receiver operating characteristics curve analysis of IQGAP3 urinary cell-free NA expression in bladder cancer patients and normal controls according to tumor grade IQGAP3 urinary cell-free NA levels in the BC patients with G1, G2, and G3 tumors were significantly higher than those in normal controls (PicoGreen-adjusted values) **(A)**. The AUCs for IQGAP3 in G1, G2, and G3 tumors (compared with controls) were 0.805, 0.915, and 0.929, respectively **(B-D)**. IQGAP3 urinary cell-free NA levels in the BC patients with G1, G2, and G3 tumors were also significantly higher than those in normal controls (RiboGreen-adjusted values) **(E)**. The AUCs for IQGAP3 in G1, G2, and G3 tumors (compared with controls) were 0.742, 0.880, and 0.885, respectively **(F-H)**. AUC, area under the curve. ^*^: *P*<0.001, ^#^: *P*=0.001

**Figure 8:**
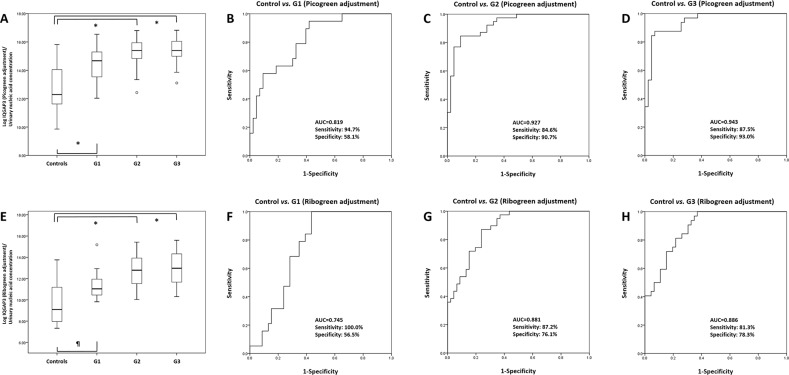
Receiver operating characteristics curve analysis of IQGAP3 urinary cell-free NA expression in bladder cancer patients and those with hematuria according to tumor grade IQGAP3 urinary cell-free NA levels in BC patients with G1, G2, and G3 tumors were significantly higher than those in hematuria controls (PicoGreen-adjusted values) **(A)**. The AUCs for IQGAP3 in G1, G2, and G3 tumors (compared with controls) were 0.819, 0.927, and 0.943, respectively **(B-D)**. IQGAP3 urinary cell-free NA levels in BC patients with G1, G2, and G3 tumors were also significantly higher than those in hematuria controls (RiboGreen-adjusted values) **(E)**. The AUCs of IQGAP3 for G1, G2, and G3 tumors (compared with controls) were 0.745, 0.881, and 0.886, respectively **(F-H)**. AUC, area under the curve. ^*^: *P*<0.001, ¶: *P*=0.002

IQGAP3 urinary NA levels were significantly higher in BC patients at all tumor grades (PicoGreen-adjusted: (all, P<0.001) and RiboGreen-adjusted values: G1 (*P*=0.001), and G2 and G3 (*P*<0.001)) than in normal controls. ROC curve analysis revealed that the AUCs for IQGAP3 at tumor grades G1, G2, and G3 (PicoGreen-adjusted) were 0.805, 0.915, and 0.929, respectively, whereas the RiboGreen-adjusted AUCs were 0.742, 0.880, and 0.885, respectively (Figure 7). In addition, IQGAP3 urinary NA levels were significantly higher in BC patients at all tumor grades (PicoGreen-adjusted: (all, *P*<0.001) and RiboGreen-adjusted: G1 (*P*=0.002), and G2 and G3 (*P*<0.001)) than in hematuria controls. ROC curve analysis revealed that the AUCs for PicoGreen-adjusted IQGAP3 urinary NAs in BC patients with tumor grades G1, G2, and G3 were 0.819, 0.927, and 0.943, respectively, whereas those for RiboGreen-adjusted IQGAP3 were 0.745, 0.881, and 0.886, respectively (Figure 8).

## DISCUSSION

The results of the present study suggest that urinary levels of IQGAP3 cell-free NA are a valuable diagnostic marker for BC. Thus, IQGAP3 urinary NA levels could be a suitable non-invasive diagnostic tool for distinguishing those with BC from those with non-cancer-associated hematuria.

The two main sources of urinary cell-free NAs are renal filtration of cell-free NAs from the blood or direct contact with the urinary tract [9]. However, urinary NA or DNA markers for urologic cancer cannot come from blood and most likely are transported directly into the urine. Hoque et al [10] detected methylated GSTP1 and RASSF1A genes in 65% of urine samples from patients with renal cancer, but in only 11% of serum samples from the same patients. Accordingly, cancer-specific urinary NAs might be shed directly into the urine by urologic cancers. Because the bladder is a temporary reservoir for urine, it is an ideal source of biomarkers for BC. Recent studies report that urinary NAs could be potential candidate diagnostic markers for BC. Casadio et al [7] identified urine cell-free DNAs derived from c-Myc, BCAS1, and HER2 in 51 BC patients, and reported a high AUC upon ROC curve analysis, suggesting that urine cell-free DNA could be a potential marker for early non-invasive BC. Our own previous study examining cell-free RNA revealed significantly higher levels in BC patients, again suggesting that they may be a valuable diagnostic marker for BC [11]. Urinary NA can be detected easily using non-invasive methods; therefore, we suggest that urinary NAs may be ideal biomarkers for BC.

Isoleucine glutamine motif-containing GTAase-activating proteins (IQGAPs) are well conserved in organisms spanning yeasts to humans [12]. IQGAPs play roles in cell adhesion, cell migration, and extracellular signaling. IQGAP1 plays a role in cancer progression, whereas IQGAP2 seems to act as a tumor suppressor [13, 14], and IQGAP3 promotes cancer growth and metastases [15]. Yang et al [15] reported that expression of IQGAP3 is markedly increased in lung cancer tissues at both the mRNA and protein levels, and that overexpression of IQGAP3 promotes tumor cell growth, migration, and invasion by modulating EGFR-ERK signaling. In addition, Qian et al [16] showed that plasma levels of IQGAP3 protein are significantly higher in those with hepatocellular carcinoma than in normal controls, suggesting that IQGAP3 may be a novel biomarker for hepatocellular carcinoma screening and diagnosis. Here, we found that tissue expression of IQGAP3 was markedly higher in BC patients than in normal controls (a result validated by real-time PCR of mRNA derived from BC and normal control tissue microarrays), and that levels of urinary IQGAP3 cell-free NA were higher in BC patients than in normal controls (a result consistent with that obtained from real-time PCR). Furthermore, we identified that urinary levels of IQGAP3 cell-free NA were higher in BC patients than in patients with hematuria (AUC = 0.910 (PicoGreen-adjusted) and AUC = 0.854 (RiboGreen-adjusted)).

The volume and concentration of urine produced by humans fluctuates markedly depending on the time of collection, diet, hydration status, the presence of diabetes mellitus, renal disease, and pituitary disease, and the use of medications such as diuretics. Hanke et al [17] showed that urinary RNA levels fluctuate markedly between morning, midday, and evening. Schmidt et al [18] showed that quantitative assessment of CK20 might serve as a non-invasive method of identifying patients with BC. They used TATA box-binding protein (TBP) in both tissue and urine as a reference gene. However, they made no comment about the value of urinary TBP as a housekeeping reference gene. Casadio et al [7] did not use a reference gene in their study of urinary cell-free DNA as a potential diagnostic marker for BC. Thus, future studies should seek to identify and validate reference genes for urinary cell-free NA. Here, we used PicoGreen and RiboGreen-based quantifications as references to quantify total cell-free NA levels in urine. PicoGreen- and RiboGreen-based quantification of total cell-free NA in urine should make it easier to normalize expression of each urinary cell-free NA to that of a housekeeping gene. In our previous study, we tried to normalize expression of urinary cell-free RNA against GAPDH; however, we found that expression of GAPDH in urine was inconsistent, making it unsuitable for such a role [11].

An advantage of the present study is the real-time PCR-based validation of increased expression of genes identified in tissue microarrays. In BC, urinary cell-free NAs might be excreted directly by cancer tissues in the form of exosomes. Thus, increased expression of certain genes in cancer tissues might be reflected by increased expression in the urine. Another advantage is that we compared PicoGreen- and -RiboGreen-adjusted expression of urinary cell-free NAs. No previous study has directly compared PicoGreen- and RiboGreen-adjusted expression of urinary cell-free NAs.

There are several limitations in this study. First, the expression of IQGAP3 also increased in lung cancer tissue and associated with poor prognosis [15]. Unfortunately, there was no experiment to explain the tissue specificity of urinary NAs of IQGAP3. Further studies are necessary to confirm the tissue specificity of NAs of IQGAP3 in BC. And although this study compared directly PicoGreen-and RiboGreen-adjusted expression of urinary cell-free NAs, there was no study which adjustment of urinary NAs is appropriate. Additional studies are necessary to confirm which adjustment of urinary NAs is appropriate. Second, because there were no results of cytology, we didn't compare the results of cytology and urinary NAs. Third, there was no validation study to confirm as a diagnostic marker in another cohort. Additional validation studies with another cohort patients with cytology are necessary.

In conclusion, IQGAP3 urinary cell-free NAs in BC patients were significantly higher than those in normal controls or in patients with hematuria. High expression of IQGAP3 urinary cell-free NAs in BC reflects high expression in BC tissue. Therefore, IQGAP3 urinary cell-free NAs may be a complementary diagnostic biomarker for BC.

## MATERIALS AND METHODS

### Study populations and samples

A total of 81 patients (74 BC and 7 normal controls; bladder trauma patients) were used for the tissue set, and 212 patients (92 BC and 120 normal controls) were used for the urine set. For the urine set, the age-adjusted controls comprised 66 healthy individuals (that visited the hospital for medical check-ups) and 54 patients with microscopic hematuria due to non-malignant conditions. Any patients diagnosed with concomitant carcinoma in situ (CIS) and pyuria were excluded. Urine and tissue samples were collected and stored as described previously [11, 19]. Tumors were staged and graded according to the 2002 American Joint Committee on Cancer TNM classification system and the 1973 WHO grading system, respectively. The methods used for sample collection and analysis were approved by the Ethics Committee of Chungbuk National University Hospital. All subjects provided written informed consent (IRB approval number: 2010-12-010).

### RNA extraction from tissues and synthesis of cDNA

RNA was extracted from BC tissues using TRIzol reagent (Invitrogen, Carlsbad, CA, USA), as described previously [8, 19], and cDNA was synthesized from 1 μg of total RNA using a first strand cDNA synthesis kit (Amersham Biosciences Europe GmbH, Freiburg, Germany), according to the manufacturer's protocol.

### Extraction of cell-free NAs from urine

Urinary cell-free NAs were extracted using the QIAamp Circulating Nucleic Acid Kit (Qiagen GmbH, Hilden, Germany). Briefly, each frozen urine sample (1 ml) was thawed at room temperature, treated with QIAGEN Proteinase K (125 μl), ACL buffer (1.1 ml), carrier RNA (5.6 μl), and ATL buffer (250 μl), and mixed by pulse-vortexing for 30 sec. The tube was then incubated at 60°C for 30 min. ACB buffer was added to the lysate and mixed thoroughly by pulse-vortexing for 15–30 sec. The lysate-ACB buffer mixture was incubated for 5 min on ice, and the lysate was applied to the tube extender of a QIAamp Mini column. The lysate was then drawn through the column under vacuum. Next, ACW1 buffer (600 μl) was applied to the QIAamp Mini column and drawn through under vacuum. This was repeated using ACW2 buffer (750 μl) and 100% ethanol. Finally, the QIAamp Mini column was centrifuged at 14000 rpm for 3 min. The assembly was then incubated at 56°C for 10 min to dry the membrane completely. After placement into a clean 1.5 ml elution tube, AVE buffer (50 μl) was added to the QIAamp Mini column membrane at room temperature for 3 min. The column was again centrifuged at 14000 rpm for 1 min to elute the NAs. Cell-free NAs were dissolved in EB buffer and stored at −20°C until use.

### Real-time PCR

Expression of urinary cell-free NAs was measured by real-time PCR using a Rotor Gene 6000 instrument (Corbett Research, Mortlake, Australia), as described previously [11]. The sequences of the gene-specific primers used for real-time PCR are listed in Table 7. All samples were run in triplicate. For the tissue studies, expression of all identified genes was normalized to that of GAPDH. For the urine studies, the Quant-iT RiboGreen RNA Reagent and Kit (Molecular probes, Eugene, OR, USA) and the Quant-iT PicoGreen dsDNA Reagent and Kit (Molecular probes, Eugene, OR, USA) were used as references to measure the concentration of total cell-free NAs purified from urine samples.

**Table 7:** Primers used in the study

Gene	Tissue mRNA expression		Urinary cell-free NAs	
**Primer (5’-3’)**	**Size (bp)**	**Primer (5’-3’)**	**Size (bp)**
CDC20	S: ATC AGA AAG CCT GGG CTT TG	175	-	-
	AS: GA AGG AAT GTA ACG GCA GGT			
IQGAP3	S: ATG AAC GCC TCA CAG CTG A	188	S: TCC ATG CAG CTG TTC TTG CC	87
	AS: AAA ACA GTG GCC TAG CTT GG		AS: CAG CAC TGG GAT TCT GCA AG	
TOP2A	A: ATG CTG CGG ACA ACA AAC AA	150	S: GAC TGT CTG TTG AAA GAA TC	88
	AS: TGA GAG CTG GGA CAT ACA TC		AS: ATT CCA CAG AAC CAA TGT AG	
UBE2C	S: GCT ACA GCA GGA GCT GAT GA	179	-	-
	AS: CTG GCA TTT GGA GAA ACA GT			

S, sense; AS, anti-sense.

### Statistical analysis

Expression of urinary cell-free NAs in the BC and control groups was compared using nonparametric methods because the data were not normally distributed and could not always be transformed to achieve normality. Receiver operating characteristic (ROC) curves were constructed and used to evaluate the diagnostic performance of the candidate urinary cell-free NAs, and the optimal cut-off points for candidate marker selection were based on the highest combined sensitivity and specificity values obtained from ROC curve analysis. The diagnostic value of the urinary cell-free NAs was determined using univariate binary logistic regression models. The BC patient microarray data set (165 BC patients versus 68 controls; 58 normal looking bladder mucosa surrounding cancer, 10 normal bladder mucosa) is available from the NCBI Gene Expression Omnibus public database (microarray data, GSE13507) [20]. Statistical analysis was performed using IBM SPSS Statistic ver. 21.0 (IBM Corp. Armonk, NY, USA) and a *P*-value <0.05 was considered significant.

## References

[1] Antoni S, Ferlay J, Soerjomataram I, Znaor A, Jemal A, Bray F (2017). Bladder cancer incidence and mortality: a global overview and recent
trends. Eur Urol.

[2] Em M, Wein AJ KL, Novick AC, Partin AW, Peters CA (2007). Urothelial tumors of the bladder.

[3] Lotan Y, Roehrborn CG (2003). Sensitivity and specificity of commonly available bladder tumor
markers versus cytology: results of a comprehensive literature review and
meta-analyses. Urology.

[4] van der Aa MN, Steyerberg EW, Sen EF, Zwarthoff EC, Kirkels WJ, van der Kwast TH, Essink Bot M (2008). Patients’ perceived burden of cystoscopic and urinary
surveillance of bladder cancer: a randomized comparison. BJU international.

[5] Simon MA, Lokeshwar VB, Soloway MS (2003). Current bladder cancer tests: unnecessary or
beneficial?. Crit Rev Oncol Hematol.

[6] Kim WT, Cho NH, Ham WS, Lee JS, Ju HJ, Kwon YU, Choi YD (2009). Comparison of the efficacy of urine cytology, Nuclear Matrix Protein
22 (NMP22), and Fluorescence in Situ Hybridization (FISH) for the diagnosis of
bladder cancer. Korean J Urol.

[7] Casadio V, Calistri D, Tebaldi M, Bravaccini S, Gunelli R, Martorana G, Bertaccini A, Serra L, Scarpi E, Amadori D, Silvestrini R, Zoli W (2013). Urine cell-free DNA integrity as a marker for early bladder cancer
diagnosis: preliminary data. Urol Oncol.

[8] Yun SJ, Yan C, Jeong P, Kang HW, Kim Y, Kim E, Lee O, Kim WT, Moon S, Kim IY, Choi Y, Kim WJ (2015). Comparison of mRNA, protein, and urinary nucleic acid levels of S100A8
and S100A9 between prostate cancer and BPH. Ann Surg Oncol.

[9] Bryzgunova OE, Laktionov PP (2015). Extracellular nucleic acids in urine: sources, structure, diagnostic
potential. Actanaturae.

[10] Hoque MO, Begum S, Topaloglu O, Jeronimo C, Mambo E, Westra WH, Califano JA, Sidransky D (2004). Quantitative detection of promoter hypermethylation of multiple genes
in the tumor, urine, and serum DNA of patients with renal cancer. Cancer Res.

[11] Kim WT, Jeong P, Yan C, Kim YH, Lee IS, Kang HW, Kim YJ, Lee SC, Kim SJ, Kim YT, Moon SK, Choi YH, Kim IY (2016). UBE2C cell-free RNA in urine can discriminate between bladder cancer
and hematuria. Oncotarget.

[12] Briggs MW, Sacks DB (2003). IQGAP1 as signal integrator: Ca2+, calmodulin, Cdc42 and the
cytoskeleton. FEBS letters.

[13] White CD, Erdemir H, Sacks DB (2012). IQGAP1 and its binding proteins control diverse biological
functions. Cell Signal.

[14] Schmidt VA, Chiariello CS, Capilla E, Miller F, Bahou WF (2008). Development of hepatocellular carcinoma in Iqgap2-deficient mice is
IQGAP1 dependent. Mol Cell Biol.

[15] Yang Y, Zhao W, Xu Q, Wang X, Zhang Y, Zhang J (2014). IQGAP3 promotes EGFR-ERK signaling and the growth and metastasis of
lung cancer cells. PLoS One.

[16] Qian EN, Sy Han, Ding SZ, Lv X (2016). Expression and diagnostic value of CCT3 and IQGAP3 in hepatocellular
carcinoma. Cancer Cell Int.

[17] Hanke M, Kausch I, Dahmen G, Jocham D, Warnecke JM (2007). Detailed technical analysis of urine RNA-based tumor diagnostics
reveals ETS2/urokinase plasminogen activator to be a novel marker for bladder
cancer. Clin Chem.

[18] Schmidt J, Propping C, Siow W, Lohse Fischer A, Toma M, Baldauf Twelker A, Hakenberg OW, Wirth MP, Fuessel S (2016). Diagnostic and prognostic value of bladder cancer-related transcript
markers in urine. J Cancer Res Clin Oncol.

[19] Kim WT, Kim J, Yan C, Jeong P, Choi SY, Lee OJ, Chae YB, Yun SJ, Lee SC (2014). S100A9 and EGFR gene signatures predict disease progression in muscle
invasive bladder cancer patients after chemotherapy. Ann Oncol.

[20] Kim WJ, Kim EJ, Kim SK, Kim YJ, Ha YS, Jeong P, Kim MJ, Yun SJ, Lee KM, Moon SK, Lee SC, Cha EJ, Bae SC (2010). Predictive value of progression-related gene classifier in primary
non-muscle invasive bladder cancer. Mol Cancer.

